# The Role of Organic Materials in Shaping the Content of Trace Elements in Iron-Contaminated Soil

**DOI:** 10.3390/ma18071522

**Published:** 2025-03-28

**Authors:** Mirosław Wyszkowski, Natalia Kordala

**Affiliations:** Department of Agricultural and Environmental Chemistry, University of Warmia and Mazury in Olsztyn, Łódzki 4 Sq., 10-727 Olsztyn, Poland; natalia.kordala@uwm.edu.pl

**Keywords:** iron pollution, humic acids, soil, microelements

## Abstract

Iron contamination negatively affects how plants grow and develop, and it has an analogous influence on the health of other organisms. The use of different types of organic soil amendments can be a strategy to reduce the effects of excess iron stress and limit its assimilation by plants. The aim of this experiment was to investigate the possibility of using organic material in the form of humic acids (HAs) to reduce the influence of iron contamination on the content of trace elements (TEs) in the soil. The content of iron in the soil increased linearly (by 14%) as more iron was added. The addition of humic acids to the soil also promoted an increase in soil Fe content (by 12%) in comparison to the series without HAs. The highest dose of iron resulted in a decrease in Cd (by 49%), Pb (by 29%), Cr (by 13%), and Zn (by 10%) and an increase in Mn (by 6%), Cu (by 16%), and Co (by 33%) in the soil in comparison to the object without Fe. However, the first dose of iron increased the lead content, and the first and second dose of Fe also increased the Zn content in the soil. The nickel content in the soil also increased to 500 mg Fe kg^−1^ of soil. Thereafter, a decline was observed in the nickel content. The addition of organic material had a different influence on the content of individual TEs in the iron-contaminated soils. The most evident constraining impact of HAs pertained to the level of Cd (reducing it by 14%) and Zn in the soil (only for two of its doses). The content of other TEs in the soil after the addition of organic material was found to be higher than in the series without HAs. This was especially evident for elements such as cobalt (Co) and lead (Pb).

## 1. Introduction

Soil is an active component of the natural environment. It is formed at the interface between the atmosphere, lithosphere, hydrosphere, and biosphere [1]. Soil plays an important role in sustainable development. It supports important social and ecosystem services [2]. Soil health is a term used to describe the state in which soil is able to function continuously and optimally as a living ecosystem to sustain plant growth, maintain or improve environmental quality, and support human health [3]. Soil health is determined by its properties (chemical, physical, and biological), as well as soil management practices and environmental conditions [4]. Healthy soil is determined by optimal levels of essential nutrients for plants, including iron (Fe). Iron in soil occurs as different compounds and in two states of oxidation (Fe^2+^ and Fe^3+^), but it can only be readily taken up and utilized by plants in its reduced form (Fe^2+^) [5]. The Fe availability in soil solutions is chiefly determined by the soil’s redox and pH potential [6]. Iron is an essential trace element (TE) for the plant life cycle [7], playing a key role in vital biological processes (e.g., photosynthesis, cellular respiration, nucleotide synthesis [8,9], and nutrient transport [10]). It is also essential for chlorophyll synthesis, and its deficiency results in increased susceptibility of plants to infection by fungal pathogens [11]. However, in excess, iron has negative effects on growth and plant biomass increase, photosynthetic efficiency [7], organic compound and nitrogen metabolism, mitochondrial respiration, and enzyme protein activity [12], and it induces oxidative stress by generating toxic hydroxyl radicals from peroxides and oxygen suboxides in the Fenton reaction [13]. These radicals cause oxidation of cellular macromolecules (proteins, nucleic acids, membrane lipids) and are uniquely damaging because plants lack their effective scavengers [14]. High doses of iron reduce plant height and shoot number [7,15], cause leaf browning and necrosis [16], inhibit seed germination [17], and increase plant sterility [12]. It has been shown that under conditions of iron excess in rice, grain yield can be reduced by up to 30%, and under particularly severe conditions, complete crop failure can occur [18,19]. Under such conditions, not only morphophysiological changes attributed to yield are observed but also changes at the molecular and genetic level [14,20]. As reported by Müller et al. [20], the expression of genes encoding, among others, ferritin (a protein responsible for iron storage) and glutamate synthase (an enzyme involved in photorespiration) is then increased, while the expression of genes encoding nicotianamine synthetase (a protein required for phytosiderophore synthesis) is decreased.

Iron toxicity mainly affects acidic sulphate and clay soils [13], as well as sandy soils with low cation exchange capacity (CEC) or those containing Fe in easily reducible form and high organic matter in moderately reducible form [21]. The soluble Fe content of soils also increases under wetland conditions [8] due to the influx of this element from upland slopes [12] and the impact of industrial effluents [22]. In addition, human activities such as steel and textile industries, tanneries, pigment and paint production, and sewage sludge disposal contribute to the increased release of Fe to the environment [12,23]. Poor water management, anaerobic conditions, and low pH and soil fertility [8] lead to a reduction of Fe^3+^ to Fe^2+^ and promote the accumulation of Fe^2+^ in the root zone [24]. Under these conditions, plants increase their uptake of iron from the soil solution and store it in their leaves, resulting in direct Fe toxicity [25].

The use of various organic soil additions can be a strategy to reduce the effects of excess iron stress and limit its assimilation by plants. One such amendment is humic acids (HAs), which some authors [26,27] have shown to be efficacious in the remediation of soil and water resources to prevent environmental degradation. HAs are organic macromolecules containing various functional groups (including phenolic, hydroxyl, and carboxyl groups) that determine their properties and the variety of environmental functions they perform [28]. It is evident that humic compounds play a pivotal role in all soil processes, thereby exerting a considerable influence on the various properties of the soil. In the natural environment, HAs increase soil water-holding capacity, improve soil structure, influence the biological cycling of elements [29], regulate soil buffering properties and redox potential, and are involved in ion exchange processes and nutrient delivery to higher plants [30]. By providing an attractive source of energy, carbon, and nitrogen for soil microorganisms, HAs increase soil biological activity [31] and thus accelerate the degradation of soil contaminants [29]. Humic substances have the ability to control the solubility and migration of trace elements (TEs) via exchange sorption, surface precipitation, complexation, chelation [32], or redox reactions [33]. Following these processes, the mobility, bioavailability [34,35], and ecotoxicity of TEs [36,37] in soil are reduced. At the same time, HAs show stronger chelating properties towards transition metals than towards other metals [38]. In addition, by entering plant tissues and inducing various biochemical effects, HAs stimulate plant growth hormone activity and increase photosynthetic pigment synthesis and tolerance to external stressors [39]. Studies on maize [40] and wheat [41], among others, have shown that the application of HAs increases total plant yield. The addition of HAs also improves root, leaf, and shoot growth [42] and facilitates seed germination in several crop species [39]. Environmentally, on the other hand, humic acids increase soil carbon sequestration [43], are a good synergist for mineral fertilizers [44,45], and can be a substitute for fossil fuel-derived cement additives, thereby reducing the carbon footprint [46].

Soil degradation is a very important and topical global problem, as it already affects one third of the Earth’s surface [43]. Soil contamination by TEs, on the other hand, is one of the most common threats (37.3%) [46]. Once the geochemical background level is exceeded, these elements negatively affect crop yield and quality [47], disrupt natural nutrient cycling, and reduce the intensity of microbial transformation of organic matter, resulting in reduced soil fertility [48]. Therefore, rapid and cost-effective soil remediation strategies and the use of environmentally friendly adsorption materials are crucial. One such promising approach is the use of HAs, whose application, as we indicated above, has a multifaceted effect.

Consequently, a study was conducted in which the impact of iron contamination on the content of trace elements in soil was mitigated by the utilization of humic acid, an organic material.

## 2. Materials and Methods

### 2.1. Pot Vegetation Experiment

Vegetation pot experiments were conducted on light soil (granulometric composition of loamy sand) [49] taken from the arable soil layer. The detailed soil properties are contained in Table 1.

**Figure 1:**
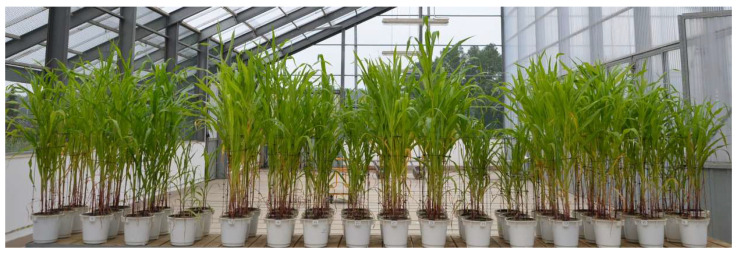
Pot vegetation experiment.

The two-factor experiment was conducted in a vegetation hall (northeastern Poland). The initial investigated factor was soil contamination with iron at concentrations of 0, 250, 500, and 750 mg kg^−1^ soil, applied as FeCl_3_. In addition, 476, 952, and 1428 mg of chlorine per kg of soil were added with FeCl_3_. The next factor was the addition of HAs to the soil at concentrations of 0, 0.3, 0.6, and 0.9 g kg^−1^ soil. The characteristics of humic acids were included in a previously published paper on HAs [50]. The test plant was maize (*Zea mays* L.). The HAs were introduced in two terms: initially and at the stage of 5 maize leaves (BBCH 15). Concurrently, the soil in all the pots was enriched with nitrogen, phosphorus, and potassium, thus ensuring the fundamental nutritional requirements of the maize crop. The nutritional composition of the nutrient solution per kilogram of soil comprised the following: 160 mg N (urea and ammonium nitrate solution—UAN, 32% N), 60 mg P (SuperFosDar 40 41.2% P_2_O_5_), and 170 mg K (potassium chloride 60% K_2_O) per kg of soil. The above additives were then mixed with the soil and introduced into 9 kg of polyethylene pots. The experiment was conducted in a controlled environment, with maize sown in the prepared soil and cultivated at a density of six plants per pot, with three replicates of each treatment. The plants were irrigated with deionized water to ensure a consistent soil moisture content of 60% of the maximum water capacity. The environmental factors during the experiments were as follows (average per month): insolation 218.1–335.6 h, air humidity 65–75%, and air temperature 11.8–21.2 ° C. The harvesting of the maize took place at the stage of panicle emergence (BBCH 59), and soil samples were collected for subsequent laboratory analysis (Figure 1).

### 2.2. Analytical Methods

**Figure 2:**
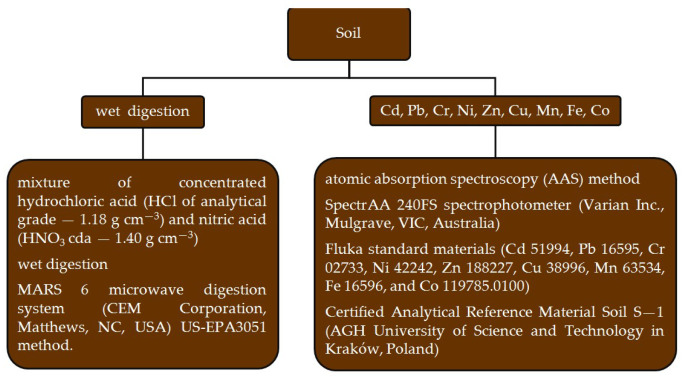
Methods of soil analysis.

Samples of soil were subjected to air-drying and sieving prior to wet digestion for trace element analysis in accordance with the US-EPA3051 method [51]. The comprehensive range of the soil analyses and characteristics of the used analytical methods [52] are presented in Figure 2.

### 2.3. Statistical Methods

The following were used to calculate the significance of differences between the factors studied: two-factor ANOVA and Tukey’s HSD test (*p* ≤ 0.01), simple correlation coefficients (** *p* ≤ 0.01 and * *p* ≤ 0.05), and relative effect of factors on the content of trace elements in the soil [53]. Calculations were performed using Statistica software version 13.3 [54]. Correlations between trace elements in the soil were calculated using the Statistica package, with all of the obtained results being taken into account.

The relative effect of factors on the content of trace elements in the soil (η^2^) was calculated for each TE using the following formula [53]:

η^2^ = SS factor/SS Total SS × 100%

where:

η^2^—relative impact of factors,
SS factor—sum of squares for a given factor,
Total SS—sum of squares for all factors.

## 3. Results

### 3.1. Iron

The presence of iron in the soil had a considerable impact on the quantity of iron present (Table 2). The content of iron in the soil increased linearly as more iron was added, up to a maximum of 14% more than in the control. The application of increasing doses of HAs resulted in elevated content of Fe in soil samples. After application of the highest dose of organic material, Fe content increased by 12% in comparison to the control series (without HAs).

### 3.2. Other Trace Elements

The effect of increasing iron doses on the other TEs content in the soil depends on the type of element (Table 3 and Table 4).

The highest dose of iron (750 mg Fe kg^−1^ of soil) led to a significant reduction in soil contamination, with Cd dropping by up to 49%, Pb by 29%, Cr by 13%, and Zn by 10% compared to the control. The first dose of iron (250 mg Fe kg^−1^ of soil) increased the Pb content in the soil by 16%. The first (250 mg Fe kg^−1^ of soil) and second dose (500 mg Fe kg^−1^ of soil) increased Zn accumulation up to 18% in comparison to the control. Iron contamination of the soil also increased Mn by 6%, Cu by 16%, and Co by 33% in the soil. The nickel content in the soil also increased to 500 mg Fe kg^−1^ of soil. Thereafter, a decline was observed in the nickel content.

The addition of HAs to the soil had different effects on the content of individual TEs in the iron-contaminated soil (Table 3 and Table 4). The most pronounced limiting effect of organic material was clearly observed for Cd, which decreased by 14% in comparison to the control series. The organic material had a similar effect on Zn, but only at two doses. The content of other TEs in the soil after HAs addition, especially Co and Pb, was higher than in the control series (without organic matter).

### 3.3. Relations Beetwen Heavy Metals

Significant correlations were found between several TEs in the soil (Table 5). An investigation was conducted into the correlation between the Fe content of soil and its Cd content, as well as its Ni, Cu, Mn, and Co contents. The findings revealed a negative correlation between Fe content and Cd content and a positive correlation between Fe content and the Ni, Cu, Mn, and Co contents. Additionally, we identified positive and highly significant correlations between Ni content and Cu, Mn, and Co; between Cu content and Mn and Co; and between Mn content and Co.

**Figure 3:**
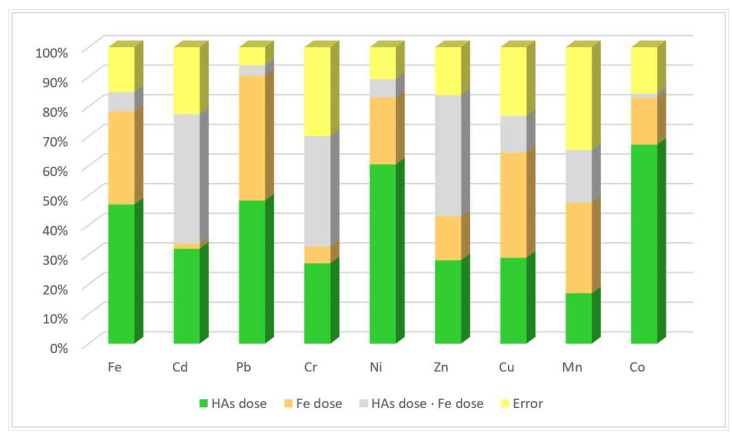
Relative impact of factors on the content of TEs in soil (percent).

The observed interaction of the factors tested in the experiment indicates that organic material in the form of humic acids had a stronger effect than soil iron pollution on the TEs content in the soil (Figure 3). The cumulative effect of soil iron contamination was higher only for Cu 35.50% and Mn 30.58%, and high for Pb 42.15% and Fe 31.43%. Organic material application had a stronger effect on Co 67.16%, Ni 60.46%, Pb 48.28%, Fe 46.94%, Cd 32.01%, and Cr 27.09%. It was also significant for the Cu content of 28.97%. There was also a very strong effect of soil contamination with iron in the interaction with organic material application on the Cr content of 37.31%, Zn 40.87%, and Cd 43.70%.

## 4. Discussion

The present study definitively shows that soil pollution with iron at the highest dose (750 mg Fe kg^−1^ of soil) limits the contents of Cd, Pb, Cr, and Zn, while there is an inverse relationship for Mn, Cu, and Co. Nickel content also increased with increasing iron dose, but only up to a dose of 500 mg Fe kg^−1^ of soil. The observed changes in trace element content may be attributable to the antagonistic effects of Fe cations towards other cationic denutrients, mainly Mn, Zn, and K. Increases in soil Fe contamination are usually linked to decreases in the availability of Mn, Zn, and K and increases in the availability of aluminum (Al), molybdenum (Mo), and Cr [55]. Kicińska and Wikar [56] assessed the degree of soils contamination near plants involved in the production of metallurgical and steel products, among others. They found that these soils had the highest Fe content (21,431.5 mg kg^−1^ d.m.), as well as Cr (39 mg kg^−1^ d.m.) and Ni (28 mg kg^−1^ d.m.). However, they also found that the levels of Zn, Mn, and Pb were lower in these soils. This is similar to the results of the present experiment. The FeCl_3_ used in this study as a source of iron also contributed to the introduction of Cl^−^ ions into the soil, thereby accelerating soil acidification and loss of alkali ions [57] and increasing soil salinity [58]. This, in turn, may have affected the bioavailability of some of the trace elements analyzed, leading to the increased Cu, Co, and Mn contents noted in this study. As reported by Bartkowiak et al. [58], chloride ion complexes are often more mobile than free trace element cations.

The reduced content of some TEs in Fe-contaminated soils may be due to the high sorption capacity of iron oxides, which leads to their immobilization by: (1) isomorphic substitution of Fe ions by divalent or trivalent cations, (2) cation exchange reactions, and (3) oxidation at the oxide surface [59]. Fazekašová and Fazekaš [60] also found elevated levels of Cd, Cr, Cu, mercury (Hg), and arsenic (As) in soil from the Nizna Slana area in Slovakia, with the primary source of pollution identified as an iron ore (siderite) mine. The accumulation of TEs may be due to the low soil pH (mean pH 5.7) and moderate organic matter content (mean 3.3%). As reported by the authors, soil pH was significantly correlated with Hg, Pb, and Cu contents, thereby suggesting a strong influence of pH on the distribution of these elements within the soil matrix.

The TE content and mobility in soils is influenced by a number of factors, including (1) the nature of the physical and chemical forms of their occurrence, (2) topography, and (3) soil properties such as pH, sorption capacity, granulometric composition (especially the amount of clay fraction), organic matter content, and oxidation–reduction potential [61]. The most significant factor influencing the bioavailability of TEs is the soil pH [62]. The pH value has a significant influence on the reactions occurring under soil conditions, including ion dissolution and co-precipitation processes and redox processes [63]. The reduction of soil pH has been demonstrated to enhance the mobility of positively charged trace element ions. This phenomenon can be attributed to the disruption of bonds between these ions and both organic and inorganic compounds within the soil matrix [64], as well as the reduction in the number of binding sites on soil colloids [65]. The effects of pH on changes in mobility and bioavailability of TEs in contaminated soils were studied by, among others, Kicińska et al. [66], showing a statistically significant positive correlation between soil acidification and the mobility of Cd, Zn, and Pb in soil samples.

There are few recently published papers on the effects of an above-optimal Fe content on the content of other TEs in the soil under controlled conditions. This can be inferred indirectly from the content of TEs in the tissues of plants grown under conditions of increased Fe supply. As shown by Müller et al. [20], high Fe levels in rice resulted in the accumulation of Cu and Co both in leaves and roots, while the opposite trend was observed for Mn and Zn. These observations suggest that iron competes with Mn and Zn in the soil solution, thereby reducing the bioavailability of these elements to plants. Our study partially confirms the observations of other authors. On the other hand, the differences observed may be due to a different soil granulometric composition or chemical form of iron used in this study.

In our study, the application of HAs to iron-contaminated soil reduced Cd and Zn contents. The usefulness of humic acids (HAs) in the phytoremediation of contaminated mine soils was also demonstrated by Vargas et al. [67]. The introduction of HAs into the soil reduced the availability of Zn and Cu by reducing the amount of their easily soluble forms. The addition of HAs resulted in an increase in the soil pH (7.6 versus 5.9), which could also contribute to the reduced mobility of these elements. In their experiment, Hattab et al. [68] carried out assisted phytostabilization of contaminated soils using sewage sludge compost and ramial chipped wood. The authors showed that the humic acids contained within the organic soil amendments significantly affected the speciation of TEs, reducing the soil mobility (availability for plants) of Pb, Cu, and Mo. In our previous study [35], the application of HAs at a rate of 0.15 g kg^−1^ had a significant effect on the soil TE content, with the effect depending on the soil kind and the type of nitrogen fertilizer. In the sand samples containing humic substances (HSs), the addition of HAs resulted in a significant decrease in various metals, including Zn (by 15%), Ni (by 19%), Pb (by 29%), Cr (by 52%), and Cd (by 58%). In the loamy sand, the accumulation of Zn (by 28%), Co (by 28%), Cd (by 34%), and Cr (by 63%) was reduced. The deprotonation of functional groups, predominantly carboxyl and phenolic groups, in HSs has been observed to occur in both neutral and alkaline environments [69]. This process has been shown to enhance the CEC of the soil, thereby affecting the speciation of TEs within the soil matrix. This, in turn, has the capacity to regulate their migration and phyto-availability [70].

The stability of the HS–TE complexes varies for different TEs. For example, complexes with Hg, Cd, or Pb are characterized by high stability, while those with Zn are less stable [71]. The positive effect of soil application of HSs (20 g dm^−3^), reducing the biologically available Cu and Pb contents in contaminated soil, was shown by Burlakovs et al. [72]. Under these conditions, after 4 months, the authors recorded an increase in the amount of stable forms of Cu (80% vs. 42%) and Pb (81% vs. 63%). Furthermore, the content of the exchangeable fraction of Cu and Pb also decreased by more than 60% for both elements. The interaction of trace element cations with humic substances is primarily facilitated via their surface oxygen-containing functional groups (-COOH, -OH, C=O), with a less significant contribution from N-containing or S-containing functional groups [73]. The bond between the trace element and the HS ligand can be formed via electron pair commonality or electrostatic attraction [74]. Another possibility is the association of a trace element with several functional groups, leading to the formation of mono- or polycyclic structures and complexes with exceptional persistence, known as chelates [75].

The presence of highly complex and relatively persistent (mineralization-resistant) organic substances such as HSs in soils significantly affects the geochemistry of TEs and their migration in the soil–plant system [76,77]. The most important property of HSs is their high sorption capacity and negatively charged reactive surface, which competes with other rhizosphere components in adsorption/chemosorption reactions of TEs [70]. Importantly, the humic acids present in HSs form more stable complexes with TEs than fulvic acids (FAs), which form soluble organometallic complexes and can increase the mobility and availability of TEs in the soil [78]. This assertion is corroborated by a study conducted by Pérez Esteban et al. [79], which demonstrated that the addition of sheep and horse manure (60 t ha^−1^) to contaminated mining soils reduced the content of the exchangeable fraction of Cu by 71% and Zn by 75%. In contrast, the pine bark series showed a different trend, characterized by an augmentation in the content of the elements analyzed that exceeded 30%. The observed differences can be attributed to the varying contents of HAs and FAs in the used organic additives.

Soils with above-optimal iron levels pose a serious environmental problem due to progressive chemical degradation and contamination by other TEs. According to a study by Saha and Bauddh [80], soils from an iron ore mine that the researchers decided to subject to assisted phytostabilization were characterized not only by elevated Fe, Pb, Cu, and Ni contents (2017.17, 65.34, 34.02, and 69.15 mg kg^−1^, respectively), but also by low pH, reduced organic matter and organic carbon, and low water-holding capacity. Therefore, the introduction of additional organic matter into such contaminated soils can improve their physicochemical properties and reduce the bioavailability of contaminants [81]. In this respect, humic acids, as amphiphilic redox compounds with unique chelating properties [70], are useful in immobilizing TEs and limiting their movement to deeper layers of the soil profile.

The present study is a significant addition to the existing literature on the potential application of humic compounds. It expands our understanding of their use and provides new information about their efficacy in making iron-contaminated soils safer. The findings of this research may be used to develop cost-effective, efficient, and environmentally friendly remediation methods for polluted soils.

## 5. Conclusions

The content of TEs in the soil depended both on the iron contamination of the soil and on the HAs application. The content of iron in the soil increased linearly (by 14%) as more iron was added. The addition of humic acids to the soil also promoted an increase in soil Fe content (by 12%) in comparison to the series without HAs.

The highest dose of iron resulted in a decrease in Cd (by 49%), Pb (by 29%), Cr (by 13%), and Zn (by 10%) and an increase in Mn (by 6%), Cu (by 16%), and Co (by 33%) in the soil in comparison to the object without Fe. However, the first dose of iron increased the lead content, and the first and second dose of Fe also increased the Zn content in the soil. The nickel content in the soil also increased to 500 mg Fe kg^−1^ of soil. Thereafter, a decline was observed in the nickel content.

The addition of organic material had a different influence on the content of individual TEs in iron-contaminated soils. The most evident constraining impact of HAs pertained to the level of Cd (reducing it by 14%) and Zn in the soil (only at two of its doses). The content of other TEs in the soil after the addition of organic material was found to be higher than in the series without HAs. This was especially evident for elements such as cobalt (Co) and lead (Pb).

## Figures and Tables

**Table 1:** Soil properties before the start of this research.

Parameter	Content
pH value in 1 M KCl dm^−3^	6.51
Cation exchange capacity—CEC (mmol + kg^−1^ DM)	82.83
Total organic carbon—TOC (g kg^−1^ DM)	3.183
Total nitrogen (g kg^−1^ DM)	0.313
Available form of (mg kg^−1^ DM):	
P	128.8
K	112.0
Mg	49.55
Total Fe (g kg^−1^ DM)	10.46
Total other TEs (mg kg^−1^ DM):	
Cd	0.241
Pb	15.86
Cr	45.36
Ni	14,20
Zn	21.10
Cu	6.556
Mn	163.9
Co	2.081

TEs—trace elements, DM—dry matter.

**Table 2:** Contents of iron, cadmium, and lead in soil, mg kg^−1^ DM.

Fe Dose mg kg^−1^ of Soil	Humic Acids (HAs) Addition in g kg^−1^ of Soil				
	0	0.3	0.6	0.9	Average
Iron					
0	10,549 *^a^*	11,326 *^a–c^*	11,918 *^cd^*	12,241 *^c–e^*	11,509 *^A^*
250	10,751 *^ab^*	12,037 *^c–e^*	12,287 *^c–e^*	12,627 *^de^*	11,926 *^B^*
500	11,754 *^b–d^*	12,142 *^c–e^*	12,768 *^de^*	12,721 *^de^*	12,346 *^C^*
750	12,064 *^c–e^*	12,641 *^de^*	13,089 *^e^*	12,732 *^de^*	12,632 *^C^*
Average	11,280 *^A^*	12,037 *^B^*	12,516 *^B^*	12,580 *^C^*	12,103
*r*	0.965	0.965	0.997	0.876	0.996
Cadmium					
0	0.229 *^d^*	0.183 *^a–d^*	0.127 *^a–b^*	0.121 *^a^*	0.165 *^A^*
250	0.225 *^d^*	0.187 *^a–d^*	0.117 *^a^*	0.157 *^a–d^*	0.172 *^A^*
500	0.203 *^cd^*	0.183 *^a–d^*	0.135 *^a–c^*	0.199 *^b–d^*	0.180 *^A^*
750	0.117 *^a^*	0.211 *^d^*	0.155 *^a–d^*	0.189 *^a–d^*	0.168 *^A^*
Average	0.194 *^B^*	0.191 *^B^*	0.134 *^A^*	0.167 *^B^*	0.171
*r*	−0.884	0.767	0.817	0.902	0.348
Lead					
0	9.05 *^a–b^*	10.60 *^b–e^*	12.09 *^c–f^*	12.95 *^e–g^*	11.17 *^C^*
250	10.49 *^b–e^*	12.57 *^d–g^*	14.91 *^gh^*	16.69 *^h^*	13.67 *^D^*
500	6.69 *^a^*	8.86 *^a–b^*	10.08 *^b–d^*	13.63 *^f–g^*	9.82 *^B^*
750	6.39 *^a^*	6.38 *^a^*	9.47 *^bc^*	11.35 *^b–f^*	8.40 *^A^*
Average	8.16 *^A^*	9.60 *^B^*	11.64 *^C^*	13.66 *^D^*	10.76
*r*	−0.776	−0.804	−0.668	−0.453	−0.701

The data are presented as the mean values. The r-value indicates the strength of the correlation. Significance at *p* ≤ 0.01 is indicated by different letters (*a–h* or *A–D*) to the right of the results.

**Table 3:** Contents of chromium, nickel, and zinc in soil mg kg^−1^ DM.

Fe Dose mg kg^−1^ of Soil	HAs Addition in g kg^−1^ of Soil				
	0	0.3	0.6	0.9	Average
Chromium					
0	54.34 *^a–d^*	50.70 *^a–c^*	54.71 *^a–d^*	56.96 *^b–d^*	54.18 *^A^*
250	54.30 *^a–d^*	55.36 *^b–d^*	56.96 *^b–d^*	55.47 *^b–d^*	55.52 *^A^*
500	53.00 *^a–d^*	55.54 *^b–d^*	61.25 *^c–d^*	52.68 *^a–c^*	55.62 *^A^*
750	47.31 *^a–b^*	54.81 *^b–d^*	63.81 *^d^*	43.66 *^a^*	52.40 *^A^*
Average	52.24 *^A^*	54.10 *^A^*	59.18 *^B^*	52.19 *^A^*	54.43
*r*	−0.865	0.705	0.993	−0.925	−0.450
Nickel					
0	7.66 *^a^*	13.84 *^c–d^*	13.97 *^c–d^*	15.07 *^c–e^*	12.64 *^A^*
250	9.24 *^a–b^*	15.44 *^c–f^*	14.00 *^c–d^*	17.04 *^d–f^*	13.93 *^A^*
500	14.22 *^c–e^*	15.62 *^d–f^*	16.88 *^d–f^*	19.01 *^f^*	16.43 *^B^*
750	11.84 *^bc^*	15.76 *^d–f^*	16.65 *^d–f^*	17.88 *^ef^*	15.53 *^B^*
Average	10.74 *^A^*	15.17 *^B^*	15.38 *^B^*	17.25 *^C^*	14.63
*r*	0.783	0.859	0.877	0.808	0.857
Zinc					
0	20.59 *^a–e^*	17.50 *^ab^*	22.13 *^c–e^*	23.83 *^d–f^*	21.01 *^B^*
250	21.65 *^b–e^*	16.93 *^a^*	26.67 *^f^*	21.48 *^b–e^*	21.68 *^B^*
500	24.23 *^ef^*	18.91 *^a–c^*	20.19 *^a–e^*	17.87 *^a–c^*	20.30 *^B^*
750	18.46 *^a–c^*	18.65 *^a–c^*	19.67 *^a–d^*	17.84 *^a–c^*	18.66 *^A^*
Average	21.23 *^BC^*	18.00 *^A^*	22.17 *^C^*	20.26 *^B^*	20.41
*r*	−0.205	0.747	−0.562	−0.950	−0.839

Significance at *p* ≤ 0.01 is indicated by different letters (*a–f* or *A–C*) to the right of the results. See Table 1 for other explanations.

**Table 4:** Contents of copper, manganese, and cobalt in soil, mg kg^−1^ DM.

Fe Dose mg kg^−1^ of Soil	HAs Addition in g kg^−1^ of Soil				
	0	0.3	0.6	0.9	Average
Copper					
0	5.331 *^a^*	5.444 *^a–c^*	5.859 *^a–c^*	5.919 *^a–c^*	5.638 *^A^*
250	5.339 *^a^*	5.912 *^a–c^*	6.538 *^a–c^*	6.070 *^a–c^*	5.965 *^A^*
500	6.198 *^bc^*	6.032 *^a–c^*	6.666 *^a–c^*	6.153 *^a–c^*	6.262 *^A^*
750	6.108 *^a–c^*	6.439 *^a–c^*	6.688 *^a–c^*	6.153 *^a–c^*	6.347 *^A^*
Average	5.744 *^A^*	5.957 *^A^*	6.438 *^A^*	6.074 *^A^*	6.053
*r*	0.869	0.979	0.862	0.918	0.973
Manganese					
0	155.5 *^a–c^*	153.2 *^ab^*	145.3 *^a^*	170.2 *^a–c^*	156.1 *^A^*
250	163.4 *^a–c^*	160.7 *^a–c^*	180.1 *^bc^*	174.7 *^bc^*	169.7 *^B^*
500	163.5 *^a–c^*	168.6 *^a–c^*	180.1 *^bc^*	175.7 *^bc^*	172.0 *^B^*
750	165.2 *^a–c^*	172.2 *^a–c^*	181.8 *^c^*	176.0 *^bc^*	173.8 *^B^*
Average	161.9 *^A^*	163.7 *^A^*	171.8 *^AB^*	174.2 *^B^*	167.9
*r*	0.867	0.989	0.799	0.883	0.888
Cobalt					
0	2.006 *^a^*	2.018 *^a–c^*	3.217 *^cd^*	3.283 *^c–e^*	2.631 *^A^*
250	2.250 *^ab^*	2.041 *^c–e^*	3.225 *^c–e^*	3.335 *^de^*	2.713 *^B^*
500	2.302 *^b–d^*	2.409 *^c–e^*	3.891 *^de^*	3.582 *^de^*	3.046 *^C^*
750	2.661 *^c–e^*	2.753 *^de^*	4.181 *^e^*	4.126 *^de^*	3.430 *^C^*
Average	2.305 *^A^*	2.305 *^B^*	3.629 *^B^*	3.582 *^C^*	2.955
*r*	0.963	0.954	0.947	0.929	0.968

Significance at *p* ≤ 0.01 is indicated by different letters (*a–e* or *A–C*) to the right of the results. See Table 1 for other explanations.

**Table 5:** Soil variable correlations.

Variable	Fe	Cd	Pb	Cr	Ni	Zn	Cu	Mn
Cd	−0.421 **							
Pb	0.191	−0.249						
Cr	0.160	−0.119	0.149					
Ni	0.792 **	−0.202	0.306 *	0.074				
Zn	−0.105	−0.305 *	0.215	0.321 *	−0.183			
Cu	0.700 **	−0.336 *	−0.056	0.215	0.530 **	0.026		
Mn	0.640 **	−0.211	0.119	0.163	0.393 **	0.132	0.541 **	
Co	0.646 **	−0.325 *	0.267	0.138	0.586 **	0.084	0.559 **	0.494 **

The results were found to be significant at * *p* ≤ 0.05 and ** *p* ≤ 0.01.

## Data Availability

The data are contained within the article.
